# Protein crystal structure determination with the crystallophore, a nucleating and phasing agent

**DOI:** 10.1107/S1600576719006381

**Published:** 2019-06-28

**Authors:** Sylvain Engilberge, Tristan Wagner, Gianluca Santoni, Cécile Breyton, Seigo Shima, Bruno Franzetti, Francois Riobé, Olivier Maury, Eric Girard

**Affiliations:** a Institut de Biologie Structurale, University Grenoble Alpes, CEA, CNRS, 71 avenue des Martyrs, CS 10090, 38044 Grenoble, France; b Microbial Protein Structure Group, Karl-von-Frisch-Strasse 10, Max Planck Institute for Terrestrial Microbiology, 35043 Marburg, Germany; c Structural Biology Group, European Synchrotron Radiation Facility, 71 Avenue des Martyrs, 38000 Grenoble, France; d Univ. Lyon, ENS de Lyon, CNRS UMR 5182, Université Claude Bernard Lyon 1, Laboratoire de Chimie, F69342 Lyon, France

**Keywords:** the crystallophore, Tb-Xo4, macromolecular crystallization, *de novo* phasing, anomalous-scattering-based methods, protein crystallography, serial crystallography

## Abstract

Obtaining crystals and solving the phase problem remain major hurdles encountered by bio-crystallographers in their race to obtain new high-quality structures. Both issues can be overcome by the crystallophore, Tb-Xo4, a lanthanide-based molecular complex with unique nucleating and phasing properties. This article presents examples of new crystallization conditions induced by the presence of Tb-Xo4. These new crystalline forms bypass crystal defects often encountered by crystallographers, such as low-resolution diffracting samples or crystals with twinning. Thanks to Tb-Xo4’s high phasing power, the structure determination process is greatly facilitated and can be extended to serial crystallography approaches.

## Abstract 2

The unique nucleating and phasing capabilities of the crystallophore, Tb-Xo4, are illustrated through challenging cases.

## 1. Introduction   

Crystallography is the method of choice for obtaining atomic scale structural information on biological macromolecules and has contributed significantly to the development of structural biology, as shown by the number of structures present in the Protein Data Bank (PDB; https://www.rcsb.org/). Crystallography has thus contributed to the understanding of protein functions, explaining complex life mechanisms and aiding in development of new drugs based on rational design.

For half a century, the method has undergone several revolutions, enabling it to maintain, during the past ten years, a PDB deposition rate of 6000–10 000 structures per year.

In a non-exhaustive way, we can quote

(i) automation of the crystallogenesis process by means of pipetting robots, allowing the use of ever smaller volumes of biological material (nanodroplet crystallization) (Santarsiero *et al.*, 2002 ▸; Brown *et al.*, 2003 ▸);

(ii) rationalization of these conditions via the numerous crystallization kits available on the market;

(iii) synchrotron light sources with their tuneable, micro(nano)-focus, automated beamlines and more recently X-ray free-electron laser (XFEL) sources;

(iv) phasing methods exploiting anomalous scattering (Hendrickson, 2014 ▸), associated, for example, with seleno­me­thio­nine labelling (Doublie, 1997 ▸) or exploitation of the intrinsic sulfur anomalous signal (Liu *et al.*, 2012 ▸; Weinert *et al.*, 2014 ▸).

In this respect, the structural genomics projects initiated in the 2000s have strongly contributed to these technological leaps. However, outcome statistics show that the success rates of the major steps in the crystal structure determination processes are low. In particular, only 30% of purified proteins will lead to crystals, half of which will allow the structure to be obtained (Terwilliger *et al.*, 2009 ▸; Khurshid *et al.*, 2014 ▸). From these structural genomics statistics, only 10% of the initial targets lead to the structure. Therefore, any new technological improvements acting on the two bottlenecks of the workflow would considerably expand the amount of new structure elucidation by crystallography. Thus, in parallel with the automation of the crystallization process, approaches favouring the nucleation stage have been developed.

Microseed matrix screening, MMS (Ireton & Stoddard, 2004 ▸), and random MMS (Shaw Stewart *et al.*, 2011 ▸) have benefited from automation and have proved to be efficient complements to conventional screening (D’Arcy *et al.*, 2007 ▸; Shaw Stewart *et al.*, 2011 ▸). However, MMS supposes that an initial reproducible condition has been determined to generate the seed stock. Another approach is the search for additives to promote nucleation and to favour crystallization. Several solid additives have been tested to promote nucleation, including mineral dusts (McPherson & Shlichta, 1988 ▸; Falini *et al.*, 2002 ▸), natural materials such as horse and human hair (D’Arcy *et al.*, 2003 ▸; Georgieva *et al.*, 2007 ▸), and carbon nanotubes (Govada *et al.*, 2016 ▸; Leese *et al.*, 2016 ▸). Porous nucleating agents appear to give the best results (Pechkova & Nicolini, 2004 ▸; Kertis *et al.*, 2012 ▸; Sugahara *et al.*, 2008 ▸; Chayen *et al.*, 2006 ▸, 2001 ▸; Saridakis *et al.*, 2011 ▸; Xing *et al.*, 2015 ▸; Khurshid *et al.*, 2015 ▸). Calixarenes (McGovern *et al.*, 2012 ▸; Rennie *et al.*, 2018 ▸; Alex *et al.*, 2018 ▸) and polyoxometalates (Bijelic *et al.*, 2015 ▸; Molitor *et al.*, 2017 ▸) have also been proposed as potential inducers of crystallization, the latter having shown phasing potential (Mac Sweeney *et al.*, 2018 ▸).

In this context, we recently proposed a lanthanide-based molecule, named the crystallophore (Tb-Xo4) (Engilberge *et al.*, 2017 ▸, 2018 ▸). Tb-Xo4 is a cationic complex with nucleating and phasing properties. These unique properties were initially highlighted on a set of eight proteins including two of unknown structure (Engilberge *et al.*, 2017 ▸). In the present study, we challenge the crystallizing and phasing properties of the crystallophore through typical crystallographic bottlenecks. In particular, we show that Tb-Xo4 may provide different crystal forms of a single protein and correct the often-encountered defects in crystalline order that may result in low-resolution data as well as twinning. We prove that, beyond these crystallization properties, Tb-Xo4 is one of the most efficient phasing tools compatible with serial crystallography approaches.

## 2. Experimental   

### 2.1. Sample preparation   

The crystallophore, Tb-Xo4, was synthetized and purified as described by Engilberge *et al.* (2017 ▸).

Two sets of protein samples were used in the present study. The first set consists of proteins overexpressed in *Escherichia coli*. This includes the protein band 9 (pb9) from phage T5 and a triple mutant (E29A-E60A-E80A) of protease 1 (PhP1) from *Pyrococcus horikoshii*. Both proteins were prepared and purified as described by Engilberge *et al.* (2017 ▸). The second set of proteins (described in Section 3.2) were directly purified from their native host *Methano­thermococcus thermolithotrophicus* [obtained as described by Wagner *et al.* (2017 ▸)]. The complete protein production and purification procedure is described in the supporting information. Four fractions, A–D, were produced, and their purity was systematically controlled by sodium dodecyl sulfate polyacrylamide gel electrophoresis (SDS-PAGE). Final pooled samples were concentrated by passing them through a 50 kDa cut-off filter, and the concentration was measured using the Bradford method with bovine serum albumin as the standard.

### 2.2. Crystallization   

Prior to crystallization, the Tb-Xo4 powder was directly solubilized with the protein sample to a final concentration of 10 m*M* according to the protocol described by Engilberge *et al.* (2017 ▸). The prepared solution was then used directly for crystallization.

Samples for *MeshAndCollect* experiments: initial crystallization conditions were determined through an automatic crystallization screening performed at the High Throughput Crystallization Laboratory of the EMBL Grenoble Outstation and optimized in 24-well plates with hanging drops set up by mixing 1.5 µl of protein solution at 10 mg ml^−1^ containing 10 m*M* Tb-Xo4 with 1.5 µl of reservoir solution (Engilberge *et al.*, 2017 ▸).

Samples resulting from shotgun purifications: crystallization screening was performed manually on 96-well two-drop MRC crystallization plates in polystyrene (Molecular Dimensions, Suffolk, UK) at 291 K. The sitting drops contained a mix of 0.7 µl of protein mixture and 0.7 µl of precipitant solution. Different screenings were performed depending on the amount of sample available. The proteins purified in fractions A and D were cocrystallized with 10 m*M* Tb-Xo4 under an anoxic tent (with a gas phase of 95% N_2_/5% H_2_). For proteins of fractions B and C, a comparative experiment was done under air in the absence and presence of 10 m*M* Tb-Xo4. For the initial screens, the JCSG+ (Molecular Dimensions) and Pentaerythritol (Jena Bioscience) kits were used for fractions A and C, the Wizard 1-4 kit (Jena Bioscience) for fraction B, and the Wizard 1-4 and Pentaerythritol kits for fraction D.

All crystallization conditions leading to crystals used in the present study are summarized in Table S1 of the supporting information.

### 2.3. Data collection, data processing and phasing   

All crystals were cryocooled in liquid nitro­gen prior to data collection, performed at 100 K (Table S1).

*MeshAndCollect* experiments: crystals were harvested using mesh-type LithoLoops of 0.40 mm diameter (Molecular Dimensions). The loop was oriented in order to get the plane of the sample holder to be perpendicular to the direction of the X-ray beam by using the mini-kappa goniometer installed on the ESRF beamline ID29 (Zander *et al.*, 2015 ▸). Crystal detection and classification were performed as described by Zander *et al.* (2015 ▸). For each crystal, partial data were collected in ±5° total rotation sections with 100 images per partial data set. Data processing and merging were performed as described by Zander *et al.* (2015 ▸) with hierarchical cluster analysis (HCA) using the newly developed software *ccCluster* (Santoni *et al.*, 2017 ▸). Data statistics are summarized in Table 1. *De novo* phasing was done in the *CRANK2* pipeline (Skubák & Pannu, 2013 ▸) without any attempt to optimize either the structure determination process or the automatic model building.

Samples purified from *M. thermolithotrophicus*: for phasing purposes with the crystallophore, crystals were used directly, *i.e.* as cocrystallized with 10 m*M* Tb-Xo4, or soaked for a short period in a solution containing 50 or 100 m*M* Tb-Xo4 (Table S1). Crystals were flash-frozen in liquid nitro­gen directly or stepwise by soaking in a cryo-preserving solution, as summarized in Table S1. Conventional macromolecular crystallography experiments were performed: diffraction data were collected on a single crystal on different beamlines at the European Synchrotron Radiation Facility (ESRF, Grenoble, France) as well as on Proxima-2A at synchrotron SOLEIL (Saint Aubin, France) as detailed in Tables 2–4 below[Sec sec3.2]. Diffraction frames were integrated using the program *XDS* (Kabsch, 2010 ▸), and the integrated intensities were scaled and merged with the programs *SCALA* and *TRUNCATE* from the *CCP4* program suite (Collaborative Computational Project, Number 4, 1994 ▸). Data statistics are summarized in Tables 2–4. Single-wavelength anomalous dispersion (SAD) phasing was performed by *AUTOSHARP* (Vonrhein *et al.*, 2007 ▸) with substructure determination via *SHELXD* (Sheldrick, 2008 ▸), with standard defaults. Molecular replacement was done with *PHASER* (McCoy *et al.*, 2007 ▸). In all cases, the *Buccaneer* software (Cowtan, 2006 ▸) was used for automated model building. Atomic models were manually improved in *COOT* (Emsley *et al.*, 2010 ▸).

Raw data used for *de novo* phasing have been made freely available on the Zenodo repository. Corresponding DOIs are listed in Table S3.

### 2.4. Refinement   

All of the models were optimized through iterative rounds of refinement and model building. Refinements were done using *BUSTER* (version 2.10.2, Global Phasing Ltd, UK). At all stages of refinement, non-crystallographic symmetry, translation–libration–screw motion and automatic water finding, as provided in *BUSTER*, were applied. After the first round of refinement, an anomalous Fourier synthesis was systematically computed to accurately place the terbium atoms.

Figures were generated with *PyMOL* (version 1.7.2, Schrödinger, LLC). Crystallographic software support was provided by SBGrid (Morin *et al.*, 2013 ▸). Refinement statistics are reported in Table S2. Structures and associated structure factor amplitudes have been deposited in the Protein Data Bank.

## 3. Results   

### 3.1. The crystallophore preserves isomorphism   

We performed two SAD phasings by merging several data sets obtained through the *MeshAndCollect* approach (Zander *et al.*, 2015 ▸; Santoni *et al.*, 2017 ▸) using protease 1 and pb9. In both cases, the beam wavelength was set to 1.6487 Å at the *L*_III_-absorption edge of terbium in order to maximize the anomalous contribution.

Within the framework of our first study (Engilberge *et al.*, 2017 ▸), we observed that samples obtained by cocrystallization with 10 m*M* Tb-Xo4 were not always sufficiently derivatized to ensure a successful phasing, even when the crystallophore induced a clear effect on the crystallization process. A soaking step in a concentrated Tb-Xo4 solution for a short period may ensure an efficient derivatization for phasing.

Therefore, the following protocol was applied: (i) crystals were grown in the presence of 10 m*M* Tb-Xo4; (ii) for data collection, crystals were soaked for 2 min in a concentrated solution of Tb-Xo4 by adding 2 µl of cryo-solution containing 100 m*M* Tb-Xo4 directly onto the crystallization drop; (iii) crystals were harvested and immediately cryo-frozen in liquid nitro­gen. Data statistics are indicated in Table 1 ▸.

#### 3.1.1. A successful *MeshAndCollect* phasing with multiple loops   

**Figure 1:**
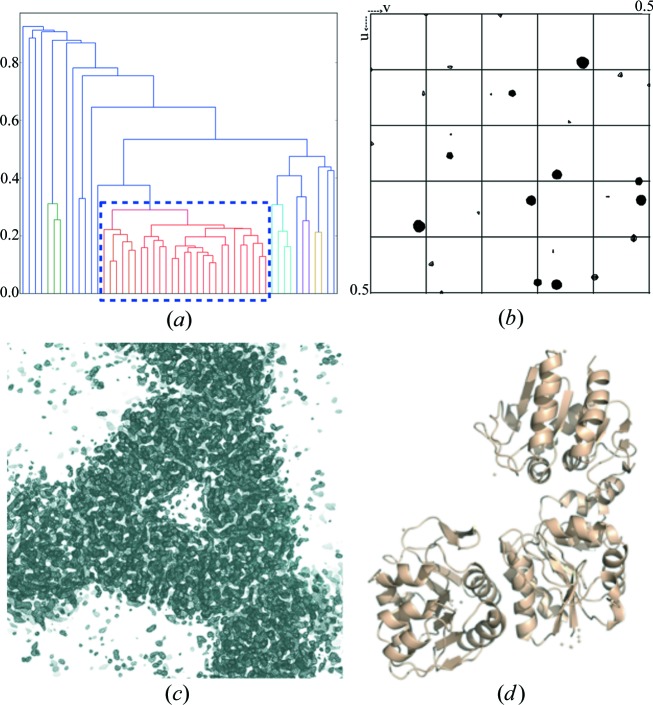
*P. horikoshii* protease 1 phasing based on data collected by the *MeshAndCollect* approach. (*a*) Dendrogram resulting from HCA on 51 sub-data sets with clustering according to correlation coefficients. The selected cluster (identified with a blue dashed rectangle) comprises 27 data sets (threshold of 0.3). (*b*) Anomalous Patterson map (Harker section *w* = 0.25). (*c*) Experimental electron density resulting from phasing with the *CRANK2* pipeline (contoured at 1σ). (*d*) Automatically built model of PhP1.

*P. horikoshii* protease 1 (PhP1) crystallization was performed in sodium malonate pH 5.5 (Table S1). Crystals with dimensions ranging from 10 × 10 × 10 µm to 30 × 30 × 30 µm were used. Data collection was performed with a beam size of 50 × 30 µm. Data were collected on four different mesh loops corresponding to four different crystallization drops, leading to 81 sub-data sets. Among them 27 were selected and merged by HCA using *ccCluster* (Santoni *et al.*, 2017 ▸) with a distance definition based on the correlation between intensities and with a coefficient linkage threshold value of 0.3 [Fig. 1 ▸(*a*)], resulting in statistics indicated in Table 1 ▸. Despite the soaking in the concentrated solution of Tb-Xo4, half of the crystals, coming from four different supports, were sufficiently isomorphous to allow the merging of their respective data sets.

Merging of data resulted in a 2.0 Å resolution data set (Table 1 ▸) with good mean *I*/σ(*I*) values (14.3 and 3.2 overall and in the highest-resolution shell, respectively) as well as excellent data indicators (CC_1/2,high_ = 88.2% and CC_1/2,overall_ = 99.7%; *R*_pim,high_ = 30.7% and *R*_pim,overall_ = 3.6%). The presence of good anomalous information, as illustrated by the clear peaks in the anomalous Patterson map [Fig. 1 ▸(*b*)], facilitated substructure determination and phasing, leading to a complete automatically built model [Fig. 1 ▸(*d*)] thanks to an easily interpretable electron density map [Fig. 1 ▸(*c*)]. The structure was then fully refined as the crystallization condition used was new (PDB code 6hf6, Table S2).

#### 3.1.2. A successful *MeshAndCollect* phasing with a low-symmetry crystal   

As mentioned in the *Introduction* [Sec sec1], Tb-Xo4 is a nucleating agent that may provide unique crystallization conditions (Engilberge *et al.*, 2017 ▸). This is what was observed in the case of pb9 with 32 unique conditions (over 576 crystallization conditions tested). The most promising conditions could be manually optimized, leading to crystals whose quality allowed facilitated phasing and automatic building of an 85% complete model from data collected to 2.0 Å resolution (Engilberge *et al.*, 2017 ▸).

In the present study, we exploited a second unique condition consisting of 10% PEG 8000, 8% ethyl­ene glycol in HEPES buffer pH 7.5 (Table S1). Prior to flash-cooling, the crystals were soaked in a cryo-solution containing 100 m*M* Tb-Xo4 and 10% PEG 8000, 25% ethyl­ene glycol in HEPES buffer pH 7.5. Crystals with average dimensions of 20 × 20 × 5 µm were soaked for 2 min as for PhP1.

**Figure 2:**
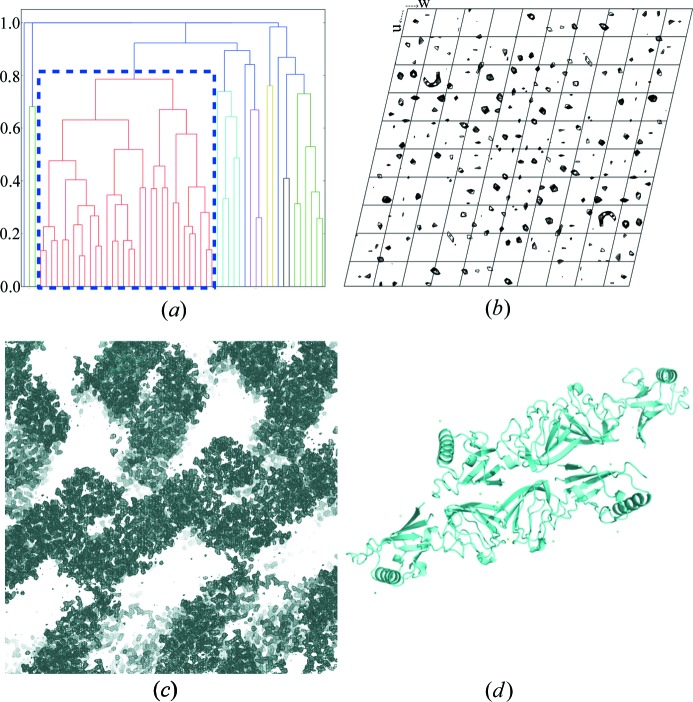
pb9 phasing based on data collected through the *MeshAndCollect* approach. (*a*) Dendrogram resulting from HCA on 55 sub-data sets with clustering according to unit-cell variation. The selected cluster (identified with a blue dashed rectangle) comprises 32 data sets (threshold of 0.8). (*b*) Anomalous Patterson map (Harker section *v* = 0.5). (*c*) Experimental electron density resulting from phasing with the *CRANK2* pipeline (contoured at 1σ). (*d*) Automatically built model of pb9.

Diffraction data were collected on a single loop using a beam size of 10 × 10 µm. Fifty-six sub-data sets were collected, and among them 32 were exploited to obtain the merged data set with statistics indicated in Table 1 ▸. In that case, the HCA was based on unit-cell variations as the metric for non-isomorphism and a coefficient linkage threshold value of 0.8 was applied [Fig. 2 ▸(*a*)]. The crystals belonged to the low-symmetry space group *P*2_1_ (Table 1 ▸). Merging yielded a 2.5 Å resolution data set with good statistics overall [mean *I*/σ(*I*) = 6.7; CC_1/2,overall_ = 97.6%; *R*_pim,overall_ = 9.5%] as well as in the highest-resolution shell [mean *I*/σ(*I*) = 3.1; CC_1/2,high_ = 78.5%; *R*_pim,high_ = 32.0%]. Despite the low-symmetry space group, the merging also led to a good anomalous signal, as illustrated by the anomalous Patterson map [Fig. 2 ▸(*b*)]. As for PhP1, the phasing resulted in a perfectly interpretable electron density map and a complete model [Figs. 2 ▸(*c*) and 2 ▸(*d*)].

### 3.2. Applications of the crystallophore approach   

**Figure 3:**
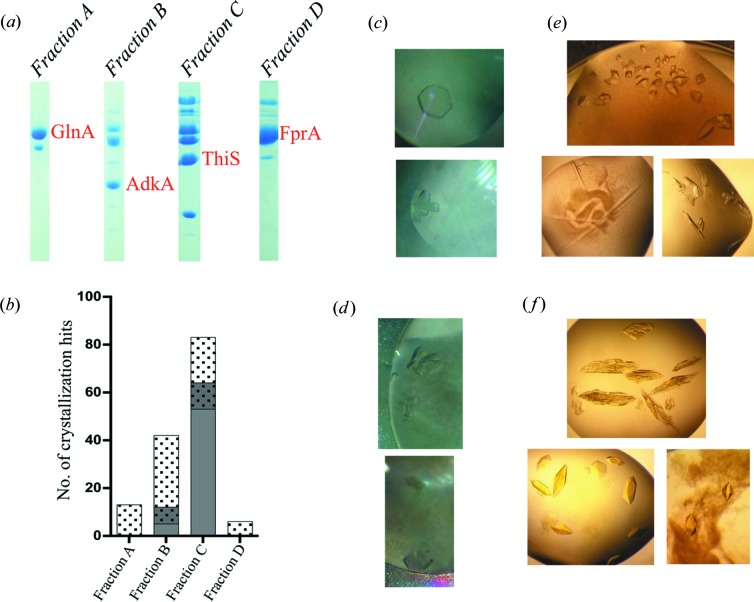
Samples obtained from *M. thermolithotrophicus* by native purification. (*a*) SDS-PAGE gels of the different purified protein fractions. (*b*) Details of the results of crystallization screening performed on the four protein fractions. The number of unique crystallization hits is depicted in grey for the native protein without Tb-Xo4 and with dots for the protein supplemented with 10 m*M* Tb–Xo4. The conditions where crystals were obtained irrespective of the presence of Tb-Xo4 are represented in grey with dots. Owing to sample quantity availability, fractions A and D were only evaluated in the presence of Tb-Xo4. (*c*)−(*f*) Examples of crystals resulting from fractions A–D, respectively. Crystallization was performed in the presence of 10 m*M* Tb-Xo4.

Next we illustrate the benefit of employing Tb-Xo4 as a routine tool in protein structure determination, using four proteins directly obtained from the marine organism *M. thermolithotrophicus*. In brief, their native purifications were obtained with four or five chromatography columns under anoxic conditions (see *Experimental* [Sec sec2] section). The resulting partially purified fractions contained two or more proteins, as determined by SDS-PAGE [Fig. 3 ▸(*a*)] and mass spectrometry.

In order to determine the crystallization conditions, the fractions were mixed with Tb-Xo4 to a final concentration of 10 m*M*. Depending on the available sample volume, several crystallization kits were evaluated. If the amount of protein sample was abundant enough, the conditions obtained in the presence of Tb-Xo4 were also compared with a native control (without the additive). More precisely, the amount of protein contained in fractions A and D allowed the screening of 96 crystallization conditions without the native control, while the abundant amount of protein in fractions B and C led to the screening of two sets of 96 crystallization conditions for each fraction which were compared with the native control (without Tb-Xo4) in both cases.

In fractions B and C, the presence of 10 m*M* Tb-Xo4 leads to unique crystallization conditions [Fig. 3 ▸(*b*)]. Depending on the protein fractions, the number of unique conditions varied from six to 30. Some of these unique conditions obtained from the first screening led to crystals that were directly exploitable (in size and diffraction quality), as illustrated in Figs. 3 ▸(*c*)–3 ▸(*f*). These crystals were used to determine the protein structures described below. As mentioned previously, the crystals obtained in the presence of 10 m*M* Tb-Xo4 were either used directly, for the most favourable cases, or soaked in a concentrated solution of Tb-Xo4 (50 or 100 m*M*) prior to cryo-preservation to facilitate the phasing procedure (Table S1).

#### 3.2.1. An example of major improvement of the crystalline quality   

Glutamine synthetase (GlnA) is a homo-dodecameric protein that plays an essential role in nitro­gen metabolism by synthesizing glutamine from ammonia and glutamic acid at the expense of ATP hydrolysis. *M. thermolithotrophicus* GlnA was obtained in fraction A [Fig. 3 ▸(*a*), left] and constituted the majority of the proteins in the fraction. Its concentration was estimated to be *ca* 15 mg ml^−1^. All attempts to determine the structure were unsuccessful as the crystals presented a mean diffraction resolution of maximum 2.60 Å as well as a significant proportion of twinning.

The addition of Tb-Xo4 resulted in crystals with a new morphology [Fig. 3 ▸(*c*)]. Recording of diffraction data on these crystals was carried out on beamline ID23-1 (ESRF, Grenoble). The addition of 10 m*M* Tb-Xo4 led to crystals diffracting up to 1.60 Å resolution. In addition, these data no longer showed twinning. They, therefore, made it possible to easily carry out a successful molecular replacement using a homologous model (PDB code 4lnf; Murray *et al.*, 2013 ▸). In this particular case, Tb-Xo4, by providing a new crystallization condition, has simultaneously solved the two issues that had hindered the GlnA structure determination. A full description of the GlnA structure and associated biology will be published elsewhere.

#### 3.2.2. The crystallophore induces two crystal forms of FprA   

The coenzyme F_420_H_2_ oxidase (FprA) is a di-iron homo-tetrameric flavoprotein catalysing the reduction of O_2_ to H_2_O (Seedorf *et al.*, 2007 ▸). FprA was isolated in fraction D [Fig. 3 ▸(*a*)] and constitutes a large proportion of the proteins in the fraction. Its concentration was estimated to be *ca* 35 mg ml^−1^. Purification and crystallization screening were performed under anaerobic conditions.

**Figure 4:**
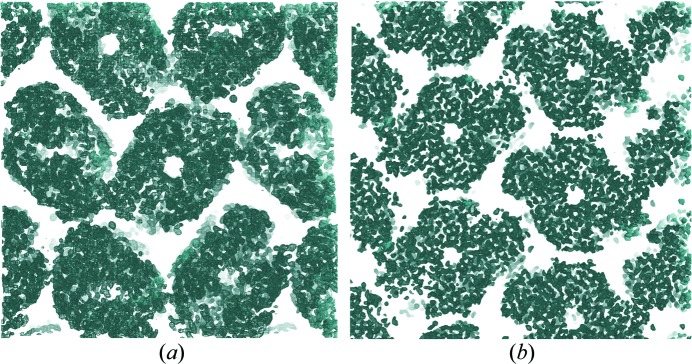
Experimental electron density map (contoured at 1σ) resulting from the SAD phasing of the data collected (*a*) at the terbium *L*_III_-absorption edge on crystal form 1 and (*b*) at a wavelength of 0.977 Å on crystal form 2 of the FprA protein.

Crystallization screening in the absence of Tb-Xo4 led to small and not well diffracting crystals. The addition of 10 m*M* Tb-Xo4 provided new crystallization conditions [Fig. 3 ▸(*b*)], two of which result in large crystals [Fig. 3 ▸(*f*) and Table S1] showing diffraction to 2.7 and 1.7 Å resolution for FprA crystal forms 1 and 2, respectively (Table 2 ▸). The two crystal forms belong to the same *P*2_1_ space group with different cell parameters and different asymmetric units, consisting of two biological tetramers and one tetramer, respectively. The presence of 10 m*M* Tb-Xo4 was sufficient in both cases for successful SAD phasing (Table 2 ▸ and Fig. 4 ▸). Despite the fact that crystal form 1 diffracted to a lower resolution and possessed a larger asymmetric unit (ASU) content (368 kDa), it was possible to extract an interpretable experimental electron density map [Fig. 4 ▸(*a*)] by collecting the data at the Tb *L*_III_-absorption edge in order to maximize the terbium anomalous contribution. In that case, 75% of the atomic model could be built automatically with the *Buccaneer* software (Cowtan, 2006 ▸). Even when data are collected far from the edge (the terbium anomalous contribution *f*′′ is about 7.5 electrons at the selenium *K*-absorption edge) as on crystal form 2, the structure (184 kDa per ASU) can be determined experimentally [Table 2 ▸ and Fig. 4 ▸(*b*)].

#### 3.2.3. A second example of multiple crystal forms induced by Tb-Xo4   

Thia­zole synthase (ThiS) is an enzyme involved in the synthesis of thi­amine diphosphate, a cofactor essential in all life forms for amino acid and carbohydrate metabolism (Zhang *et al.*, 2016 ▸). ThiS consists of a homo-octamer, as described previously for *Methano­coccus jannaschii* (Zhang *et al.*, 2016 ▸), and was isolated in fraction C [Fig. 3 ▸(*a*)], where it represents about 40% of the total protein content.

Four crystallization conditions corresponding to three different space groups were identified in the absence and presence of Tb-Xo4 (Tables S1). The observed diffraction ranges from 2.69 to 2.0 Å resolution (Table 3 ▸). The three different asymmetric units contain from four to eight protein chains, thus corresponding to 112–223 kDa, respectively.

**Figure 5:**
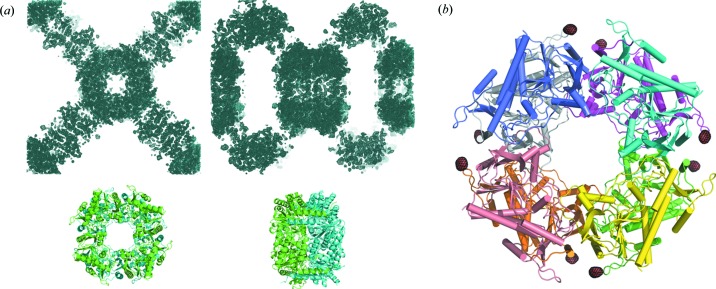
(*a*) Two orthogonal views of the experimental electron density map (contoured at 1σ) resulting from the SAD phasing of the ThiS crystal (form 1) soaked in 100 m*M* crystallophore. Cartoon representations of the ThiS biological unit are shown. (*b*) Anomalous Fourier synthesis (in red contoured at 8σ) computed with data collected on ThiS crystal form 3.

Crystal form 1 was obtained in two crystallization conditions (Table S1). Contrary to FprA which was directly phased from the 10 m*M* Tb-Xo4 added during crystallization, the co-crystallization of ThiS in the presence of 10 m*M* Tb-Xo4 was not sufficient to derive useful phases for SAD phasing from crystal form 1. We thus applied a 9 min soaking of one native crystal into a cryo-preserving solution containing 100 m*M* Tb-Xo4 (Table S1). Data collection at the Tb *L*_III_-absorption edge led to SAD phases of excellent quality, providing an interpretable experimental electron density map at 2.55 Å resolution, as illustrated by the complete model (six protein chains, 1566 amino acids in the ASU) automatically built by the *Buccaneer* software [Fig. 5 ▸(*a*)]. This example shows that Tb-Xo4 can be used as a conventional heavy atom through soaking of native crystals. Crystalline form 2 was solved by molecular replacement with *Phaser* (McCoy *et al.*, 2007 ▸) using as search model the structure of ThiS form 1. Finally, the third crystalline form, co-crystallized in the presence of 10 m*M* Tb-Xo4, was directly frozen in liquid nitro­gen and exhibited an exploitable anomalous signal. We managed to solve the structure without an extra soak of Tb-Xo4, and 80% of the atomic model could be built automatically with the *Buccaneer* software.

The model of ThiS corresponding to form 1 soaked with 100 m*M* Tb-Xo4 was fully refined (Table S2, PDB code 6hk1). The model is composed of six protein chains, and 49 Tb sites were modelled with refined occupancies ranging from 0.1 to 0.58. By using this complete refined model, we computed an anomalous Fourier synthesis for crystal form 3 and observed the fixation of one Tb per monomer [Fig. 5 ▸(*b*)].

#### 3.2.4. An example of *ab initio* phasing   

**Figure 6:**
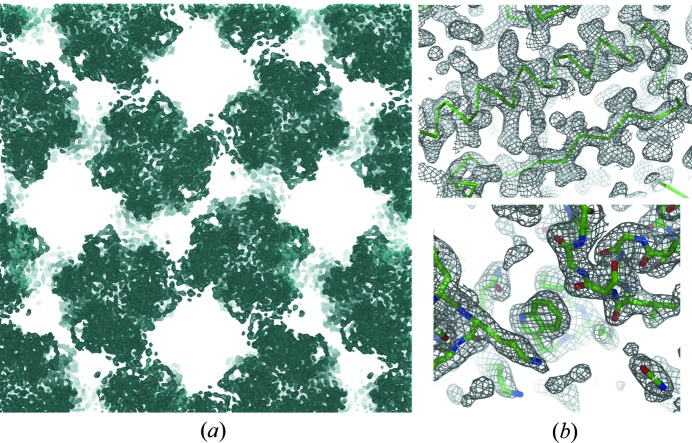
AdkA protein. (*a*) Experimental electron density map (contoured at 1σ) resulting from the *ab initio* phasing based on data collected on a crystal soaked in 50 m*M* crystallophore. (*b*) Portions of the model built automatically, leading to the identification of the AdkA protein sequence superimposed on the electron density map depicted in (*a*).

As illustrated in Fig. 3 ▸(*a*), fraction B contains several proteins. We successfully obtained different crystallization hits for this protein mixture in the presence of 10 m*M* Tb-Xo4. In the absence of the crystallophore, no crystal was obtained in the same conditions. Because of the presence of multiple proteins in this fraction, it was quite difficult to assign the protein identity to the crystal by using mass spectrometry. Consequently, to ensure the efficiency of the phasing as described in Section 3.1, we systematically soaked all the crystals in a solution containing 50 m*M* Tb-Xo4, and we collected the data at the *L*_III_-absorption edge to maximize the terbium anomalous contribution. Among the recorded data sets, one presented diffraction at 2 Å resolution and a significant amount of anomalous signal (Table 4 ▸). A tentative SAD phasing with *AutoSharp* (Vonrhein *et al.*, 2007 ▸) resulted in a perfectly interpretable electron density map, allowing a small portion of the model to be built manually (Fig. 6 ▸). The protein was sequenced using the electron density, and a search for the sequence in the genomic database of *M. thermolithotrophicus* matched with adenylate kinase (AdkA), a trimeric enzyme that catalyses the interconversion of adenine-type nucleotides (Criswell *et al.*, 2003 ▸). By providing the full sequence, the automatic building completed the model at 95%.

The model was then fully refined (Table S2, PDB code 6hf7). This led us to identify four Tb-Xo4 binding sites per trimer with refined occupancies ranging from 0.4 to 0.7.

## 4. Discussion   

The crystallophore, Tb-Xo4, is a cationic lanthanide complex having nucleating as well as phasing properties. These properties were initially demonstrated on a set of eight proteins, including two of unknown structure (Engilberge *et al.*, 2017 ▸). In particular, Tb-Xo4 allows experimental determination of the structure by means of anomalous-scattering-based experi­ments for all eight tested proteins. It also demonstrates a clear influence on the crystallization phase diagram of these proteins, by increasing the number of possible crystallization conditions and even by generating unique hits (Engilberge *et al.*, 2017 ▸).

In the present study, we challenged the crystallographic properties of Tb-Xo4 in single-crystal production and structure determination by tackling complicated cases in real-life conditions.

### 4.1. Tb-Xo4 is an efficient nucleating/crystallizing agent   

Implementation of the crystallophore during the crystallization process is quite simple. The Tb-Xo4 powder is directly solubilized with the protein solution to reach a final concentration of 10 m*M*, this concentration corresponding to the most efficient one to induce nucleation (Engilberge *et al.*, 2017 ▸). Up to now, no adverse effects, in particular protein precipitation, have been observed at this stage.

We have already shown that the crystallophore promoted unique crystallization conditions and induced protein crystal growth even at low protein concentrations (Engilberge *et al.*, 2017 ▸). This unique property may be of interest for precious samples as well as to reduce the sample quantity or to expand crystallization screenings. The present study confirms the capability of the crystallophore to induce new crystallization conditions that cannot be obtained in its absence [Fig. 3 ▸(*b*)]. All the crystals grown in the presence of Tb-Xo4 present a well ordered packing, as witnessed by the diffraction between 2.70 and 1.60 Å.

Moreover, we show that Tb-Xo4 is able to promote selective crystallization from samples containing several proteins, as illustrated by the crystallization of the protein mixture contained in the purified fractions from the marine archaea *M. thermolithotrophicus*. A recent X-ray/density functional theory analysis (Engilberge *et al.*, 2018 ▸) revealed that the number and the affinity of Tb-Xo4 binding sites is protein dependent. These observations suggest that Tb-Xo4 can favour crystallization of proteins which present appropriate crystallophore binding sites. Thus, it would be interesting to confirm the observed trend with a more systematic study.

Low-resolution diffracting samples and twinning are examples of obstacles encountered by macromolecular crystallographers in their route to structure determination. Both are related to crystal growth disorders and thus defects in the crystalline order. As exemplified by the GlnA example, the presence of Tb-Xo4 during the crystallization process may correct such defects by inducing a new crystal form diffracting at high resolution without twinning.

Finally, we have shown in two different examples that the crystallophore induced different crystal packing. For both FprA and ThiS proteins, several crystal forms were obtained. Such a property is thus of interest for studies of ligand–protein complexes and for structure-based drug design (Müller, 2017 ▸). Generating new crystal forms may avoid packing issues that limit soaking with the ligand, that hinder access to ligand binding sites or that have a distorting effect on the binding mode of the evaluated ligand.

### 4.2. Tb-Xo4 is a powerful phasing agent   

With the exception of the glutamine synthetase determined by molecular replacement, we have been able to determine the structures of the five proteins used in the present study through SAD phasing, at least in one crystal form. Phasing was greatly facilitated thanks to the large anomalous signal of the lanthanide ion, which allows structure determination of large molecular weight proteins to be tackled (Girard *et al.*, 2003 ▸; Hendrickson, 2014 ▸). This is particularly true when data are collected at the *L*_III_-absorption edge, where the anomalous contribution is maximized (with *f*′ and *f*′′ of *ca* 30 electrons). However, successful non-optimized SAD phasing, as for FprA crystal form 2, can be envisaged on fixed-wavelength synchrotron beamlines or on a laboratory X-ray source since the terbium *f*′′ contribution is *ca* 7 electrons and 9 electrons at 12.7 keV and for Cu *K*α radiation, respectively.

Moreover our approach based on lanthanide complexes reinforces the efficient exploitation of the strong anomalous signal of lanthanide ions. This approach enables various and multiple supramolecular interactions with protein residues that diversify the possible binding sites with good occupancies. The design of the crystallophore followed the same philosophy and has benefited from our past experience of deciphering the mode of interactions of lanthanide complexes at the protein’s surface (Girard *et al.*, 2002 ▸; Dumont *et al.*, 2013 ▸; Stelter *et al.*, 2014 ▸).

Recently, the lanthanide complex Gd-HPDO3A (Girard *et al.*, 2002 ▸) has been used for *de novo* phasing purposes using data collected on XFEL sources (Barends *et al.*, 2014 ▸; Gorel *et al.*, 2017 ▸). In the present study, we have demonstrated that Tb-Xo4 is perfectly compatible with serial crystallography experiments. Two successful SAD phasings were performed on data collected with the *MeshAndCollect* approach (Zander *et al.*, 2015 ▸; Santoni *et al.*, 2017 ▸), including the protein pb9 as an example of a low-symmetry crystal (Figs. 1 ▸ and 2 ▸). For both presented examples, even with soaking of the crystals in a solution containing 100 m*M* Tb-Xo4, the level of isomorphism between the crystals was sufficiently preserved and kept sufficiently high to derive useful phases.

The high phasing power of Tb-Xo4 is also clearly illustrated by the various successful structure determinations of the present study and confirms our initial work, in which two protein structures were solved *de novo* (Engilberge *et al.*, 2017 ▸). Moreover, we recently determined the structure of a 380 kDa complex composed of three proteins with Tb-Xo4 (Vögeli *et al.*, 2018 ▸), showing that the crystallophore does not promote the dissociation of such complexes. Attempts to solve the structure of the latter work using conventional heavy atoms as well as first-generation lanthanide complexes (*e.g.* Gd-HPDO3A) did not succeed. Conversely, the smooth and efficient derivatization provided by Tb-Xo4 was a success, and more than 20 Tb-Xo4 binding sites were identified (Vögeli *et al.*, 2018 ▸). The crystallophore can also be used to directly derive native crystals, as shown for ThiS as well as for the structure determination of vitamin B_12_ transporter BtuM (Rempel *et al.*, 2018 ▸) and the structure of propionyl-CoA synthase from *Erythrobacter sp.* NAP1 (Bernhardsgrütter *et al.*, 2018 ▸). Finally, the straightforward *ab initio* phasing of the protein AdkA is an additional example of the unmatched phasing power of Tb-Xo4.

For future studies exploiting Tb-Xo4 phasing properties, the following guide may be helpful. We recommend to soak the crystals in a solution containing 50–100 m*M* crystallophore to increase the occupancy of existing heavy-atom sites and promote additional ones. On the basis of our previous study (Engilberge *et al.*, 2017 ▸) and the results presented here, even if promising samples can be obtained by cocrystallization in the presence of 10 m*M* Tb-Xo4, this is not a guarantee of successful phasing: a trial and error step is generally required to assess the presence of a sufficient anomalous signal.

Finally, a comparison of the crystallophore with seleno­me­thio­nine incorporation shall be made. Seleno­me­thio­nine labelling using the recombinant approach is one of the most common methods to solve *de novo* protein structures. Despite the diversity of expression systems now available (Walden, 2010 ▸), seleno-labelled proteins can lead to artefacts (induced different conformations, non-isomorphism compared with unlabelled proteins *etc.*) or difficulties in phasing (the anomalous contribution is *ca* 10 electrons and crystals sometimes suffer from incomplete incorporation or possess disordered seleno­me­thio­nine). In this respect, Tb-Xo4 is one alternative to seleno­me­thio­nine labelling. It provides stronger anomalous power, as its anomalous contribution is three times larger than that of selenium (at their respective absorption edges).

By adding its nucleating properties to its phasing abilities, Tb-Xo4 will surely bring about a new revolution in the macromolecular crystallography field, as seleno­me­thio­nine labelling did a few decades ago.

## 5. Conclusion   

Here, we have presented new examples of the capabilities offered by the crystallophore, a cationic lanthanide complex with nucleating and phasing properties. The several examples shown in this study highlight the incomparable efficiency of Tb-Xo4 to direct structural elucidation of new proteins. This ‘all-in-one’ molecule overcomes the common bottlenecks found in macromolecular crystallography, such as the absence of crystallization, badly ordered crystal, twinning and phasing issues. Thus, it should be considered as a new efficient tool in the protein crystallographer’s toolbox which dramatically improves the structure determination process by acting on the two main bottlenecks of macromolecular crystallography, thus saving samples and time.

## Supplementary Material

Supporting information file. DOI: 10.1107/S1600576719006381/gj5222sup1.pdf

PDB reference: protease 1 from *Pyrococcus horikoshii*, 6hf6

PDB reference: thiazole synthase from *Methano­thermococcus thermolithotrophicus*, 6hk1

PDB reference: Adenylate kinase from *Methanothermococcus thermolithotrophicus*, 6hf7

Serial crystallography dataset - PhP1: https://doi.org/10.5281/zenodo.2640319

Serial crystallography dataset - pb9: https://doi.org/10.5281/zenodo.2640356

X-ray diffraction images for coenzyme F420H2 oxidase (FprA) from M. thermolithotrophicus, form 1: https://doi.org/10.5281/zenodo.2641965

X-ray diffraction images for coenzyme F420H2 oxidase (FprA) from M. thermolithotrophicus, form 2: https://doi.org/10.5281/zenodo.2641991

X-ray diffraction images for thiazole synthase from M. thermolithotrophicus: https://doi.org/10.5281/zenodo.2641868

X-ray diffraction images for adenylate kinase from M. thermolithotrophicus: https://doi.org/10.5281/zenodo.2641812

## Figures and Tables

**Table 1:** Data-processing statistics of the *MeshAndCollect* data Values in parentheses refer to the highest-resolution shell.

	Protease 1	pb9
Wavelength (Å)	1.648	1.648
Partial data sets collected	81	56
Partial data sets processed	51	55
Partial data sets merged	27	32
Space group	*P*4_1_2_1_2	*P*2_1_
Unit cell (Å, °)	*a* = *b* = 124.62; *c* = 130.31	*a* = 71.52; *b* = 95.77; *c* = 71.58
		β = 102.88
Resolution range (Å)	19.99–2.00 (2.04–2.00)	19.77–2.50 (2.60–2.50)
Total No. of reflections	1 258 634	193 660
No. of unique reflections	69 275	32 493
Completeness (%)	99.5 (99.8)	99.6 (99.6)
Multiplicity	18.2 (16.3)	6.0 (5.7)
*R*_merge_ (%)	16.0 (121.8)	21.7 (71.4)
*R*_pim_ (%)	3.6 (30.7)	9.5 (32.0)
〈*I*/σ(*I*)〉	14.3 (3.2)	6.7 (3.1)
Half-set correlation CC_1/2_	0.997 (0.882)	0.976 (0.785)

**Table 2:** Data-processing statistics obtained from the two crystal forms of the FprA protein in the presence of 10 m*M* Tb-Xo4 Values in parentheses refer to the highest-resolution shell.

	FprA	FprA
	Crystal form 1	Crystal form 2
Wavelength (Å)	1.649	0.977
Beamline	Proxima-2A (SOLEIL)	ID23-1 (ESRF)
Space group	*P*2_1_	*P*2_1_
Unit cell (Å, °)	*a* = 84.33; *b* = 148.43; *c* = 145.97	*a* = 73.66; *b* = 144.97; *c* = 74.21
	β = 90.45	β = 91.78
Resolution range (Å)	49.48–2.69 (2.84–2.69)	42.83–1.74 (1.84–1.74)
No. of unique reflections	95 828	156 345
Completeness (%)	96.6 (91.4)	99.0 (94.8)
Multiplicity	6.3 (6.3)	5.6 (5.2)
*R*_merge_ (%)	14.3 (115.4)	8.7 (96.7)
*R*_pim_ (%)	7.6 (54.2)	4.0 (45.8)
〈*I*/σ(*I*)〉	7.2 (1.4)	9.1 (1.6)
Half-set correlation CC_1/2_	0.994 (0.525)	0.998 (0.659)
SigAno†	1.141 (0.604)	0.890 (0.593)

† As calculated by *XDS*.

**Table:** Values in parentheses refer to the highest-resolution shell.

	ThiS (10 m*M* Tb-Xo4)	ThiS (soaked with 100 m*M* Tb-Xo4)
	Crystal form 1	Crystal form 1
Wavelength (Å)	1.649	1.649
Beamline	ID23-1 (ESRF)	ID23-1 (ESRF)
Space group	*I*422	*I*422
Unit cell (Å)	*a* = *b* = 216.82; *c* = 207.50	*a* = *b* = 216.86; *c* = 207.25
Resolution range (Å)	49.14–2.10 (2.21–2.10)	49.09–2.55 (2.68–2.55)
No. of unique reflections	142 478	79 324
Completeness (%)	99.4 (95.9)	98.8 (94.9)
Multiplicity	26.0 (23.6)	39.9 (39.4)
*R*_merge_ (%)	24.3 (329.4)	34.0 (337.1)
*R*_pim_ (%)	4.9 (69.7)	5.4 (53.4)
〈*I*/σ(*I*)〉	10.9 (0.8)	11.6 (1.4)
Half-set correlation CC_1/2_	0.999 (0.489)	0.998 (0.577)
SigAno†	1.117 (0.597)	1.871 (0.665)

**Table:** 

	ThiS (10 m*M* Tb-Xo4)	ThiS (10 m*M* Tb-Xo4)
	Crystal form 2	Crystal form 3
Wavelength (Å)	0.966	1.649
Beamline	ID30a (ESRF)	ID23-1 (ESRF)
Space group	*P*22_1_2_1_	*P*3_2_21
Unit cell (Å)	*a* = 73.53; *b* = 96.19; *c* = 160.14	*a* = *b* = 94.50; *c* = 405.65
Resolution range (Å)	47.19–2.00 (2.10–2.00)	47.29–2.69 (2.84 –2.69)
No. of unique reflections	77 452	59 436
Completeness (%)	99.5 (97.3)	99.8 (98.8)
Multiplicity	3.9 (3.6)	7.5 (7.3)
*R*_merge_ (%)	8.5 (94.5)	11.7 (132.1)
*R*_pim_ (%)	4.7 (55.8)	4.6 (52.1)
〈*I*/σ(*I*)〉	8.7 (1.3)	9.1 (1.2)
Half-set correlation CC_1/2_	0.998 (0.356)	0.999 (0.820)
SigAno†	1.547 (0.827)	1.921 (0.582)

† As calculated by *XDS*.

**Table 4:** Processing statistics obtained on AdkA experimental data Values in parentheses refer to the highest-resolution shell.

	AdkA (50 m*M* Tb-Xo4)
Wavelength (Å)	1.649
Beamline	ID23-1 (ESRF)
Space group	*P*42_1_2
Unit cell (Å)	*a* = *b* = 131.33; *c* = 88.40
Resolution range (Å)	48.92–1.96 (2.07–1.96)
No. of unique reflections	55 867
Completeness (%)	99.8 (99.0)
Multiplicity	24.9 (21.2)
*R*_merge_ (%)	9.5 (126.1)
*R*_pim_ (%)	2.5 (28.0)
〈*I*/σ(*I*)〉	23.1 (2.3)
Half-set correlation CC_1/2_	0.999 (0.739)
SigAno†	2.323 (0.771)

† As calculated by *XDS*.
