# Supplementary material for: Protein crystal structure determination with the crystallophore, a nucleating and phasing agent
Source: J Appl Crystallogr. 2019 Jun 28;52(Pt 4):722–31. doi: 10.1107/S1600576719006381 (PMC6662991; doi:10.1107/S1600576719006381)
Supplement: Supplementary file 1 [file j-52-00722-sup1.pdf]

JOURNAL OF APPLIED CRYSTALLOGRAPHY

Volume 52 (2019)

Supporting information for article:

Protein crystal structure determination with the crystallophore, a nucleating and phasing agent

Sylvain Engilberge, Tristan Wagner, Gianluca Santoni, Cécile Breyton, Seigo Shima, Bruno Franzetti, Francois Riobé, Olivier Maury and Eric Girard

S1. Procedure of production and purification of samples from *Methanothermococcus thermolithotrophicus*

Methanothermococcus thermolithotrophicus (DSM 2095), obtained from the Deutsche Sammlung von Mikroorganismen und Zellkulturen (DSMZ, Braunschweig, Germany) was grown at 65 °C as previously described (Wagner *et al.*, 2017).

Protein fractions have been enriched and purified using the shotgun approach as described in (Vögeli *et al.*, 2018). The purification of fractions A-D were performed under anoxic atmosphere (95% N_2/5% H_2) at 18 °C and without any freezing step. About 40 g of wet cell pellets were suspended in lysis buffer (50 mM MOPS/NaOH pH 7.0, 10 mM $MgCl_2$ and 2 mM dithiothreitol) with a ratio of 10 ml lysis buffer per g of pellet. The cells were lysed by osmotic shock at 18°C under gentle stirring with an addition of 2.0 U/ml benzonase to reduce viscosity. The lysate was centrifuged twice at 10,000 ×g for 60 min at 4 °C to remove cell debris. The supernatant was applied on a DEAE Sepharose fast-flow column (65 ml) equilibrated with 50 mM Tricine/NaOH pH 8.0 containing 2 mM dithiothreitol (DTT) (buffer A). The column was washed at a flow rate of 4.5 ml/min for 2 column volumes. The elution step was performed through a gradient of increasing concentration of NaCl from 50 mM to 350 mM in 5 column volumes. The protein fractions eluted between 130 and 240 mM NaCl were pooled and diluted with an equal volume of buffer A and then the sample was loaded on a Q-Sepharose fast-flow column (50 ml) (GE Healthcare, Freiburg) pre-equilibrated in buffer A. The column was washed with 200 mM NaCl, and the proteins were eluted at a flow rate of 3.0 ml/min with a gradient from 200 to 400 mM NaCl in 3 column volumes. Protein fractions eluted between 235 - 370 mM NaCl were pooled and diluted with an equal volume of 25 mM sodium phosphate buffer pH 7.6, containing 2 mM DTT (buffer B) and passed on a hydroxyapatite ceramic type I column (45 ml) (Macroprep; Bio-Rad; München, Germany) equilibrated with buffer B. The column was washed at 3 ml/min with buffer B with 5 column volumes and the flow-through containing the protein from fraction A and B was collected. A gradient of 25 - 500 mM of sodium phosphate was applied to the hydroxyapatite for 4 column volumes and the protein fractions eluted at 240 - 490 mM sodium phosphate pH 7.6 were collected. This latter protein pool containing the fraction C and D was subsequently diluted with three volume of 25 mM Tris/HCl, pH 7.6, containing 2.0 M $(NH_4)_2SO_4$ and 2 mM DTT (buffer C), and injected on a Source 15 Phenyl column (15 ml) that was pre-equilibrated in the same buffer. After washing the column with 1 column volume of buffer C, the elution was done at a flow rate of 1 ml/min with a gradient from 2.0 to 0 M $(NH_4)_2SO_4$ in 14 column volumes. Protein of fraction C eluted within a range of 0.90 - 0.77 M $(NH_4)_2SO_4$ and protein from fraction D eluted within the range of 0.73 - 0.60 M $(NH_4)_2SO_4$. Each fraction (hydroxyapatite flow-through containing A and B; fraction C and fraction D from Source 15 Phenyl column) was concentrated by passing them through a 50 kDa cut-off filter (Merck Millipore, Darmstadt, Germany). Ultimately, the concentrated sample was injected onto a 10/300 Superose 6 column (GE Healthcare) equilibrated in 25 mM Tris/HCl pH 7.6,

containing 10% glycerol and 2 mM DTT and eluted at a flow rate of 0.4 ml/min. The flow through fraction from the hydroxyapatite containing the protein from fraction A and B were injected on the Superose 6 and both fractions were separated by their elution volume: 10.6 - 13.6 ml for fraction A and 14.1 - 18.1 ml for fraction B. Proteins from fraction C were injected on the Superose 6 and eluted between 11.6 and 14.9 ml. Proteins from fraction D eluted between 13.6 and 16 ml on the Superose 6.

Table S1 Crystallization, soaking and cryo-preserving solutions used for the different proteins studied in the present work.

Protein acronym	Crystallization condition	Cryo-preserving solution	Soaking
pb9	10 % PEG8000<br>8 % Ethylene glycol<br>100 mM HEPES pH 7.5<br>10 mM Tb-Xo4	10 % PEG8000<br>25 % Ethylene glycol<br>100 mM HEPES pH 7.5<br>100 mM Tb-Xo4	In cryo solution<br>Soaking time 2 min
PhP1	2.8 - 3.2 M Malonate pH 5.5<br>10 mM Tb-Xo4	3.4 M Malonate pH 5.5<br>100 mM Tb-Xo4	In cryo solution<br>Soaking time 2 min
FprA form 1	20 % PEG 8000<br>200 mM Ammonium sulphate<br>10 % 2-propanol<br>100 mM HEPES pH 7.5<br>10 mM Tb-Xo4	20 % PEG 8000<br>200 mM Ammonium sulphate<br>10 % 2-propanol<br>100 mM HEPES pH 7.5<br>25 % Glycerol	None
FprA form 2	28 % PEG 400<br>200 mM Calcium chloride<br>100 mM HEPES pH 7.5<br>10 mM Tb-Xo4	28 % PEG 400<br>200 mM Calcium chloride<br>100 mM HEPES pH 7.5<br>10 mM Tb-Xo4	None
ThiS form 1	1.6 M Magnesium sulphate<br>100 mM MES pH 6.5<br>10 mM Tb-Xo4	1.6 M Magnesium sulphate<br>100 mM MES pH 6.5<br>25 % Glycerol	None
ThiS form 1 soaked with Tb-Xo4	44 % PEE 797<br>100 mM HEPES pH 8.0	44 % PEE 797<br>100 mM HEPES pH 8.0<br>100 mM Tb-Xo4	In cryo solution<br>Soaking time 9 min
ThiS form 2	28 % PEG 400<br>200 mM Calcium chloride<br>100 mM HEPES pH 7.5<br>10 mM Tb-Xo4	28 % PEG 400<br>200 mM Calcium chloride<br>100 mM HEPES pH 7.5<br>10 mM Tb-Xo4	None
ThiS form 3	45 % PEP 629<br>300 mM Potassium chloride	45 % PEP 629<br>300 mM Potassium chloride	None

	100 mM TRIS pH 8.5<br>10 mM Tb-Xo4	100 mM TRIS pH 8.5<br>10 mM Tb-Xo4	
AdkA	25 % PEP 629<br>50 mM Magnesium chloride<br>100 mM HEPES pH 7.5<br>10 mM Tb-Xo4	25 % PEP 629<br>50 mM Magnesium chloride<br>100 mM HEPES pH 7.5<br>50 mM Tb-Xo4	In cryo solution<br>Soaking time 6 min
GlnA	35 % PEE 797<br>200 mM Ammonium sulphate<br>100 mM HEPES pH 7.5<br>10 mM Tb-Xo4	35 % PEE 797<br>200 mM Ammonium sulphate<br>100 mM HEPES pH 7.5<br>10 mM Tb-Xo4	None

PEP 629 stands for Pentaerythritol propoxylate (17/8 PO/OH); PEE 797 stands for Pentaerythritol ethoxylate (15/4 EO/OH)

Table S2 X-ray refinement statistics.

	Protease 1	AdkA	ThiS form1
PDB code	6HF6	6HF7	6HK1
Resolution (Å) [a]	19.99 - 2.00 (2.08 - 2.00)	48.92 - 1.96 (2.03 - 1.96)	49.09 - 2.55 (2.64 - 2.55)
No. of reflections	69161 (6821)	55799 (5378)	79189 (7534)
Reflections used for R_{free} [b]	3384 (338)	2824 (264)	3957 (362)
R_{work}	0.162	0.171	0.189
R_{free} [b]	0.181	0.190	0.213
No. of atoms			
Proteins	3931	4494	12405
Ligand/ion	115	76	315
Water	553	550	438
B factors $(Å)^2$			
Protein	37.67	50.24	56.23
Ligand/ion	53.41	42.56	66.14
Water	56.10	55.50	61.36
R.m.s deviations [c]			
Bond lengths (Å)	0.013	0.015	0.009
Bond angles (°)	1.57	1.58	1.44
Clashscore	0.62	0.87	1.51
Ramachandran favored (%)	99.39	98.24	97.80
Ramachandran allowed (%)	0.61	1.59	2.20
Ramachandran outliers (%)	0.00	0.18	0.009

[a] Values relative to the highest resolution shell are within parentheses. [b] R_{free} was calculated as the R_{work} for 5% of the reflections that were not included in the refinement. [c] R.m.s, root mean square.

Table S3 Raw data availability.

Data set	Corresponding DOI
Protease1 *MeshAndCollect*	https://doi.org/10.5281/zenodo.2640319
pb9 *MeshAndCollect*	https://doi.org/10.5281/zenodo.2640356
FprA crystal form1	https://doi.org/10.5281/zenodo.2641965
FprA crystal form2	https://doi.org/10.5281/zenodo.2641991
ThiS crystal form3	https://doi.org/10.5281/zenodo.2641868
AdkA	https://doi.org/10.5281/zenodo.2641812
